# Clinically acceptable agreement between the ViMove wireless motion sensor system and the Vicon motion capture system when measuring lumbar region inclination motion in the sagittal and coronal planes

**DOI:** 10.1186/s12891-017-1489-1

**Published:** 2017-03-21

**Authors:** Hanne Leirbekk Mjøsund, Eleanor Boyle, Per Kjaer, Rune Mygind Mieritz, Tue Skallgård, Peter Kent

**Affiliations:** 1 Department of Sports Science and Clinical Biomechanics, University of Southern Denmark, Odense, Denmark; 2 Dalla Lana School of Public Health, University of Toronto, Toronto, Canada; 3 School of Physiotherapy and Exercise Science, Curtin University, Perth, Australia

**Keywords:** Movement measurement, DorsaVi, Lumbar spine, Assessment, Validity, Bland Altman method

## Abstract

**Background:**

Wireless, wearable, inertial motion sensor technology introduces new possibilities for monitoring spinal motion and pain in people during their daily activities of work, rest and play. There are many types of these wireless devices currently available but the precision in measurement and the magnitude of measurement error from such devices is often unknown. This study investigated the concurrent validity of one inertial motion sensor system (ViMove) for its ability to measure lumbar inclination motion, compared with the Vicon motion capture system.

**Methods:**

To mimic the variability of movement patterns in a clinical population, a sample of 34 people were included – 18 with low back pain and 16 without low back pain. ViMove sensors were attached to each participant’s skin at spinal levels T12 and S2, and Vicon surface markers were attached to the ViMove sensors. Three repetitions of end-range flexion inclination, extension inclination and lateral flexion inclination to both sides while standing were measured by both systems concurrently with short rest periods in between. Measurement agreement through the whole movement range was analysed using a multilevel mixed-effects regression model to calculate the root mean squared errors and the limits of agreement were calculated using the Bland Altman method.

**Results:**

We calculated root mean squared errors (standard deviation) of 1.82° (±1.00°) in flexion inclination, 0.71° (±0.34°) in extension inclination, 0.77° (±0.24°) in right lateral flexion inclination and 0.98° (±0.69°) in left lateral flexion inclination. 95% limits of agreement ranged between -3.86° and 4.69° in flexion inclination, -2.15° and 1.91° in extension inclination, -2.37° and 2.05° in right lateral flexion inclination and -3.11° and 2.96° in left lateral flexion inclination.

**Conclusions:**

We found a clinically acceptable level of agreement between these two methods for measuring standing lumbar inclination motion in these two cardinal movement planes. Further research should investigate the ViMove system’s ability to measure lumbar motion in more complex 3D functional movements and to measure changes of movement patterns related to treatment effects.

## Background

Although low back pain (LBP) causes more global disability than any other health condition [1], our knowledge of the relationship between movement and LBP is limited [2]. LBP is associated with movement changes such as reduced range of motion, decreased proprioception and slower movements when compared with people without LBP [2]. Uncertainty remains about whether these movement characteristics exist prior to LBP onset or are a result of LBP. Furthermore, we have limited knowledge of lumbar movement patterns when people are active in everyday living, away from clinical or laboratory settings. Measurement methods that could reliably and validly measure lumbar movement patterns in daily activities would potentially improve our knowledge of the links between LBP and movement patterns.

Measuring lumbar motion in clinical settings includes observation, Fingertip to Floor Test, Schober’s Test or measurements taken by devices such as inclinometers. However, these methods are limited to only being able to measure a static position, typically at end range and they require a clinician to be present.

In research settings, more advanced laboratory 3D analysis systems, such as the Vicon system, have been used for measuring lumbar motion [3]. Infrared cameras that detect movement of surface markers positioned on the human body and calculations based on marker movements are considered very precise with reconstruction errors of <1 mm [4]. However, such 3D analysis is time-consuming and requires a large laboratory with expensive equipment and considerable technical expertise.

New sensor and smartphone technologies based on accelerometers, gyroscopes and magnetometers have been developed to measure lumbar motion [5–10]. Unlike traditional measurement methods, these technologies have the advantages of being wearable and portable, making it possible to monitor people’s activity in everyday contexts such as in work, recreation or other activities of daily living. In addition, some of these systems are equipped with functions that allow people to self-report pain events and receive individualised biofeedback during movement. These functions provide new possibilities for collecting information on the relationship between pain and how people move in everyday life, as well as new possibilities for movement rehabilitation strategies in a patient’s activities of daily living [10, 11].

The ViMove system (previously called the Back Strain Monitor) is a wearable motion sensor system (DorsaVi.com, Melbourne, Australia) capable of measuring three dimensions of lumbosacral movements for up to 24 h, capturing data on pain reporting and providing biofeedback [11]. When measuring lumbosacral motion, the movement sensors are attached to the skin of the back and sacrum using tape.

The ViMove system has demonstrated good intra-tester (ICC(2,1) > 0.89) and inter-tester (ICC(2,1) > 0.86) reliability for lumbar range of motion in a sample of 23 healthy participants [11]. A concurrent validity pilot study compared ViMove measurements to Optotrack 3D motion analysis and found excellent accuracy with standard errors of measurement of 0.9° (95%CI = ±1.8°) for the sagittal plane and 1.8° (95%CI = ±3.6°) for the coronal plane [12]. However, that pilot study contained only two healthy participants and the concurrent validity of ViMove needs further investigation using a larger study sample with a more generalisable variability in movements and range of symptoms. Also, as the authors did not adjust for correlation between their measurements, those estimates of concurrent validity have some imprecision and should be replicated using more robust statistical methods.

Therefore, the aim of this study was to investigate the concurrent validity of ViMove motion sensors for measuring lumbar region inclination motion, using Vicon measurements as the reference standard. We compared their capacity for measuring lumbar region surface movement, rather than lumbar spine intersegmental motion, because that is what clinicians routinely assess in the clinic. Although it is often believed that treatment interventions for LBP specifically affect symptomatic structures in the lumbar spine, treatment decisions in clinical practice are based on movement patterns (lumbar region motion) visually observed by the clinician, rather than intersegmental spinal kinematics. Some clinicians believe that intersegmental spinal kinematics can be determined by skilled palpation, however the evidence indicates this is unreliable, with agreement at a level similar to chance [13–15].

An additional consideration in choosing this method was the absence of a validated Vicon kinematic model for intersegmental movement of the lumbar spine. While there are some differences between the lumbar movement patterns of people with and without pain, there is also considerable overlap between these populations [2], and as we were not aware of any a priori reasons for why the average concurrent validity might differ between these populations, we recruited participants with and without LBP to ensure a mixed study sample with diverse movement patterns. We also chose to test people rather than testing movements generated by artificial/robotic equipment because people’s spines can move in unpredictable ways.

## Methods

### Study population

All participants were recruited among students and employees at the University of Southern Denmark.

LBP was defined as pain between the lower costal margins and above the inferior gluteal folds [16]. Participants with LBP had to have current or recurrent LBP. Current LBP was arbitrarily defined as experiencing an average pain of >2 on a 0–10 Numeric pain Rating Scale (NRS) over the past 3 weeks. Recurrent LBP was defined as “LBP which has occurred at least 2 times over the past year with each episode of LBP lasting at least 24 h, with a pain intensity of >2 on an 11-point NRS (>20mm on a 100mm VAS) and with at least a 30-day pain-free period between episodes” [17]. Participants with no LBP could not have (i) experienced an episode of LBP during the past year lasting >24 h with pain intensity self-reported as >2 on an NRS, or (ii) LBP during the past 3 weeks, or (iii) been currently seeking care for LBP. All participants had to be >18 years old and able to read and communicate in Danish.

### Test procedures

Data were collected in the movement laboratory at the Department of Sports Science and Clinical Biomechanics, University of Southern Denmark, Odense campus, by a physiotherapist (the examiner) trained in the use of both the ViMove and Vicon equipment. To provide descriptive information about the study population, participants’ height and weight were measured and they self-completed a questionnaire pack that included their general demographic attributes and any LBP-related characteristics, including the Roland Morris Disability Questionnaire [18] (23-item version) and Numeric pain Rating Scale [19] (0 to 10 scale).

### Positioning of sensors and surface markers

The ViMove sensors were positioned on the lumbar spine of the participant using the following procedure. The participant was asked to stand in his/her usual standing position, while both Posterior Superior Iliac Spines were palpated and marked. A line was drawn between the top of both Posterior Superior Iliac Spines and the lower sensor was placed midline below the line. The position of the upper sensor was determined by use of one of four ViMove application templates held on top of the lower sensor, selected using the height of each participant. This procedure aims to position the lower sensor at the level of the second sacral segment (S2) and the upper sensor at the twelfth thoracic segment (T12). The ViMove sensors were attached to the participant’s skin by hypoallergenic double-sided tape.

**Fig. 1:**
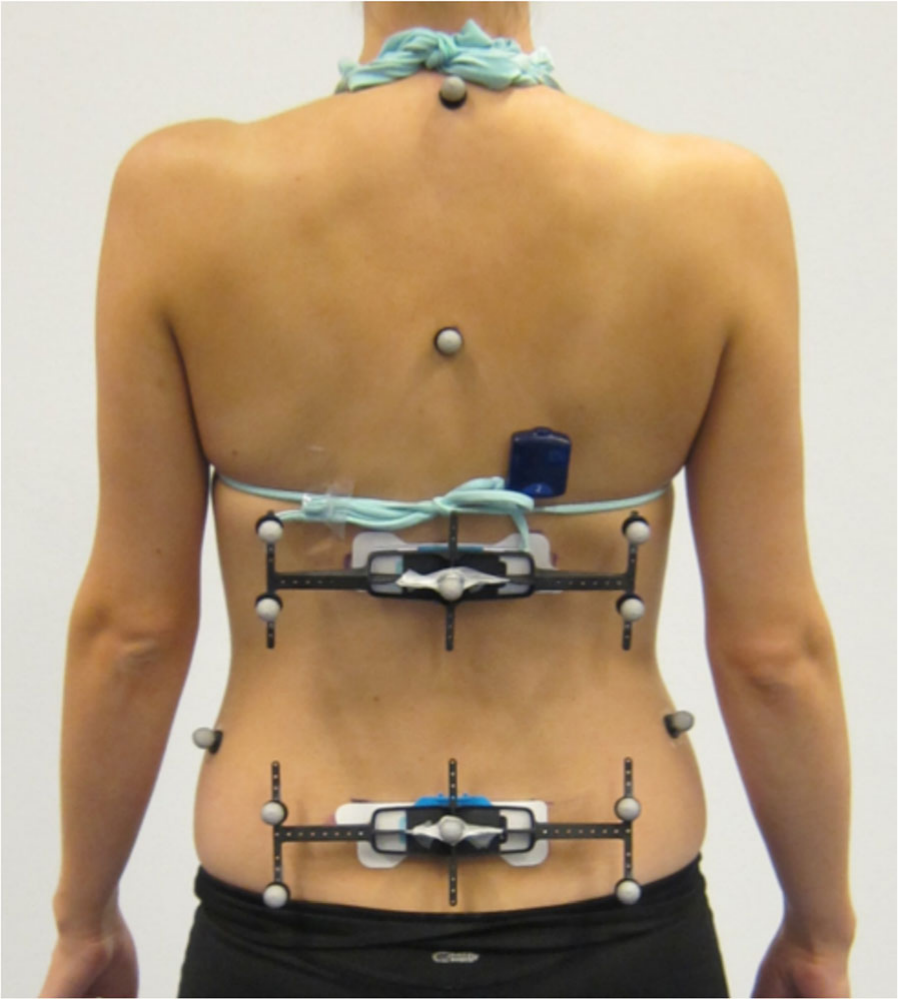
Placement of ViMove sensors and Vicon surface markers during the testing procedure

Five Vicon surface markers were mounted on each of two plastic frames that had been produced for research purposes by the DorsaVi company to make a close fit with the ViMove sensors. The frames were attached to each ViMove sensor as pictured in Fig. 1. Additional Vicon markers were attached to the Suprasternal Notch, Posterior Superior Iliac Spines bilaterally and at the level of the C7 and T5 spinous processes for use in another study and were not analysed in the current study. An accelerometer that was synchronised to the Vicon system was attached approximately 3 cm above the upper ViMove sensor.

### Movements and instructions

The neutral lumbar position for each participant was initially recorded by both systems while the participant was asked to stand still for 5 s in his/her usual standing position, and then perform three repetitions each of flexion, extension, right lateral flexion and left lateral flexion to their comfortable end of range. For each repetition, the participant was asked to move at a comfortable speed, to hold each movement position for 2 s, and to return to the standing position before the cue for a new repetition. The examiner demonstrated all test movements and standardised instructions were given for each movement direction.

Before the participant was asked to start moving, the examiner started the data capture and tapped the back of the participant, just above the upper ViMove sensor. As it was not possible to zero calibrate both systems to an identical starting point, the tap created a clear spike in the data from both systems, which was used to align the two systems.

### Equipment

The ViMove equipment consisted of two sensors, each with an integrated accelerometer, magnetometer and gyroscope, plus a Radio Frequency Device for data collection. Recordings were sampled at approximately 20 Hz and the Radio Frequency Device was connected to a computer by a USB to allow data extraction. ViMove software was used to import the data from the devices into a computer and to display these data numerically and graphically.

The Vicon system consisted of 8 MX-T20 (2 megapixel), 8 MX-T40 (4 megapixel) and 2 Bonita digital high speed cameras (1 megapixel), which were driven by Nexus software (version 1.8.5) at a sampling rate of 200 Hz (Vicon Motion Systems Inc, Los Angeles, CA, USA). Surface markers of 14 mm were used and a Myon accelerometer sampling at 3000 Hz was synchronised to the Vicon system.

### Definition of measurement angles

The ViMove system calculated the angle of the upper sensor and the lower sensor separately relative to the line of gravity. The angle between these two segments was reported automatically by the ViMove software as the lumbar angle. To calculate a comparable lumbar angle from the Vicon system, two segments were created in the Nexus Motion Capture software from the five surface markers attached to each frame and the lumbar angle was defined as the angle between these two segments. The angles were extracted from Nexus using a short software script written in the program ‘Vicon BodyBuilder’.

During the initial recording of the neutral standing position (zero position), angles for both segments relative to the line of gravity were determined concurrently for both systems and all subsequent inclination angles were reported relative to this position. For ViMove, this was a process automated within the software system. For Vicon, the average angle during 3 s of static recording was used.

### Data management

Single-plane inclinometer measurements of movements performed in the sagittal plane (flexion, extension) and coronal plane (lateral flexion) were analysed. Rotation was not assessed, as lumbar rotation range of motion is small and this version of the ViMove sensors was previously known to be imprecise in this plane of lumbar movement [12].

#### Synchronisation of data

Data management, data cleaning and all statistical analyses were undertaken using Excel for Mac 2011 version 14 (Microsoft Corporation, Redmond, WA, USA) and Stata version 13 (Stata Corp, College Station, Texas, USA). Each data set consisted of either each participant’s ViMove or Vicon data from three repetitions of one movement direction. To synchronise the data sets from a common point, the highest data spike created by the initial tap in each movement direction was identified. As the Vicon accelerometer data were sampled at 3000 Hz and Vicon angular data were sampled at 200 Hz, the angular data were then up-sampled and merged with the accelerometer data at 3000 Hz using a common frame number in the two datasets, to allow for identification of the spike in the angular data set.

#### Down-sampling

As the frequency of ViMove data was generally slightly lower than 20 Hz and not constant between recordings, the individual sample frequency for each movement direction in each participant was estimated. This was performed by identifying the spike in both recordings and identifying an identical measurement characteristic point in both the graphed datasets after the third movement repetition. Then the time between these two data points was calculated. This gave an estimation of the actual ViMove sample rate (assuming that the Vicon sample rate was constant at 200 Hz) and that sample rate was used to down-sample the Vicon data to the estimated ViMove sampling rate and allow these two sets of angular data to be merged. The mean estimated sampling frequency for ViMove was 19.6 Hz (full range 19.0–19.8 Hz).

### Statistical analysis

Descriptive data about the study population were presented as means (95% CI) or medians (interquartile range) depending on the data distribution of each variable.

#### Analysis of concurrent validity

Concurrent validity was estimated by calculating root mean squared errors between measurement methods by use of a multilevel mixed-effects linear regression model, and also by calculating mean differences and LOA using the Bland Altman method [20, 21].

In the regression models for each movement direction, the Vicon angular measurements were the dependent variable and the ViMove data were the independent variable. To account for both those variables containing multiple repeated measures for all participants, the Vicon angular measurements were the level one variable of the multilevel model and the identification numbers of the participants were the level two variable [20]. Root mean squared errors were calculated from mean errors (residuals) from the multilevel model to account for differences between the two methods having both positive and negative values.

Agreement between ViMove and Vicon was visualised using Bland Altman plots [21]. Mean differences represented the average difference between the ViMove and Vicon methods, while the LOA represented the random error or variation between methods. When investigating agreement between repeated measurements, such as in the current study, where each person had approximately 800 data points per movement direction, the traditional Bland Altman method would narrow the estimation of variance due to autocorrelation between these sequential data points [21]. The method proposed by Bland Altman (2007) was therefore used to account for both within-subject and between-subject variance using a one-way analysis of variance [21]. To minimise the influence of autocorrelation, LOA were estimated using five randomly selected data points from each participant’s data to create a reduced dataset of 170 observations from the whole dataset, and the process was repeated 100 times so that the average LOA could be reported. To visualise how differences between systems varied over time, a graph of angular measurements for each system was created for every participant in each movement direction, and a representative example is presented in the results.

As we analysed concurrent validity through the full range of movement, we use the term ‘through range’ to contrast the findings with studies that only assessed concurrent validity at end of range.

#### Sample size calculation

Precise power calculations for multilevel models require a priori estimates of variability and correlation between measurements that were unavailable for this study. Therefore, we used the rule of thumb of having a sample size that allowed between 20 and 50 clusters at the second level of the multilevel model [20] and arbitrarily chose the mid-point of 34 participants. This powered the sample for a two-level linear mixed-effect model with approximately 800 measurements per participant from each device for each movement direction.

## Results

Eighteen people with LBP and 16 people without LBP participated in the study, and their demographic data are presented in Table 1. The LBP participants’ mean pain intensity was 2.8 (95%CI 1.2 to 3.7) on a 0 to 10 Numeric pain Rating Scale [19], and mean activity limitation was 8.1 (5.7 to 10.5) (0–23 Roland Morris Disability Questionnaire) [18]. Three participants had LBP of more than 3 months’ duration.

**Table 1:** Characteristics of participants

Characteristics	All participants (*n* = 34)		
	Mean	95% CI	Full range
Age (yr)	31.2^a^	26.4 to 44.0	19 to 67
Female	47%		
Weight (kg)	77.1	71.2 to 83.1	46.7 to 132.5
Height (m)	1.74	1.71 to 1–76	1.60 to 1.86
BMI (kg/m2)	24.1^a^	22.0 to 27.8	19.7 to 36.8

^a^Median and inter-quartile range due to non-normal distribution. *BMI* body mass index

With one exception, data were available and analysed for all four movement directions for all 34 people, with an average of 784 data points for each participant in each movement direction. Extension measurements for one participant were excluded due to hypermobility, causing the Vicon marker frames to touch during their end range of motion.

**Fig. 2:**
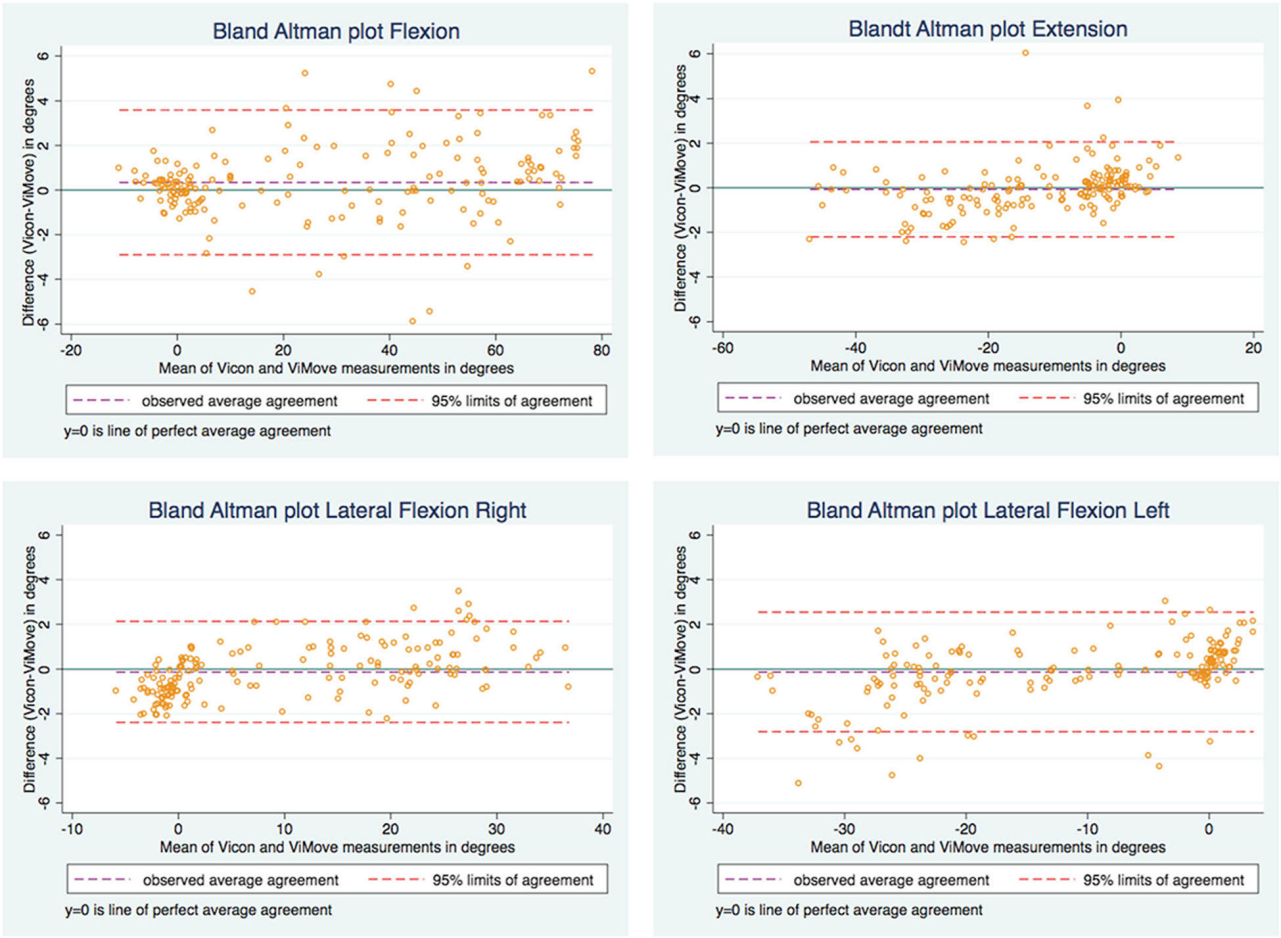
Bland Altman Plots showing the range within which 95% of the differences in measurements occurred between the ViMove and ViCon systems

**Fig. 3:**
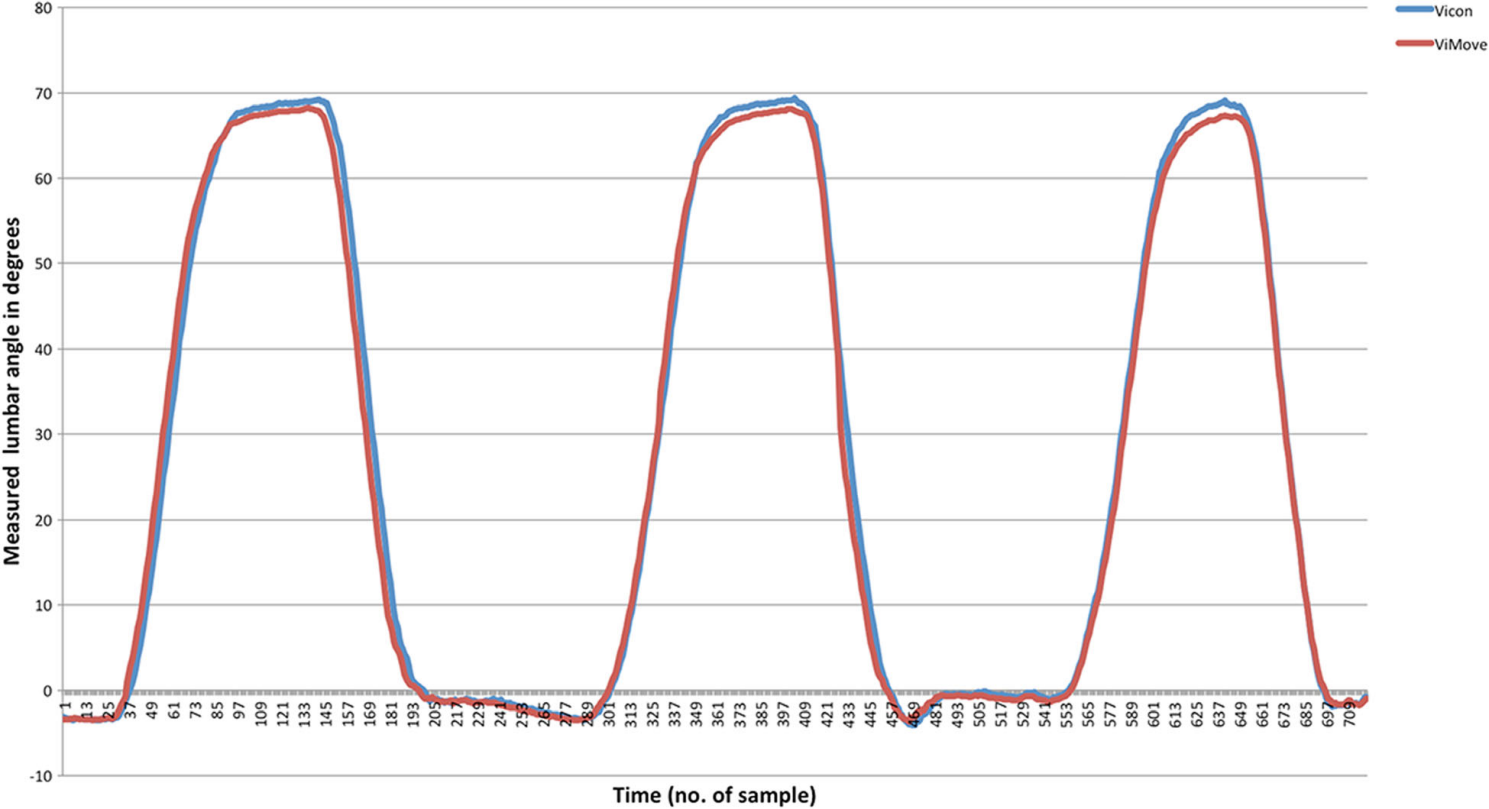
An example of measurements from both systems during three repetitions of flexion inclination by a single participant

The root mean squared errors between Vicon and ViMove measurements varied across movement directions from 0.7° to 1.8°, with the larger differences between measurements being observed in flexion inclination (Table 2). The mean difference estimated by the Bland Altman method was in the range of 0.1° to 0.4° across movement directions, which was also largest for flexion inclination. LOA showed that 95% of the differences in measurements between ViMove and Vicon would be less than 4.7° in flexion inclination, 2.2° in extension inclination, 2.4° in lateral flexion right inclination and 3.1° in lateral flexion left inclination (Fig. 2). The purple stippled line in the figure represents the mean difference between methods and the orange stippled lines are the upper and lower LOA. An example of measurements from both systems during three repetitions of flexion is seen in Fig. 3, illustrating how differences between systems could be visualised.

**Table 2:** Measurement differences between the ViMove and Vicon systems

Inclination direction	Root mean squared error (SD)	Mean difference	Lower limit of agreement	Upper limit of agreement
Flexion	1.82 ± 1.00	0.42	-3.86	4.69
Extension	0.71 ± 0.34	-0.12	-2.15	1.91
Lateral Right	0.77 ± 0.24	-0.16	-2.37	2.05
Lateral Left	0.98 ± 0.69	-0.08	-3.11	2.96

*SD* standard deviation (SD). All values are presented in degrees of movement

## Discussion

### Principal findings

The aim of this study was to investigate the concurrent validity of ViMove sensors for measuring lumbar region inclination motion, using the Vicon measurement system as the reference standard. We consider the agreement between these systems to be clinically acceptable for measuring *through range* flexion inclination, extension inclination and lateral flexion inclination, with root mean squared errors less that 2°, average differences less than 0.5°, and 95% LOA between 4.7° and -3.9°. Differences between measurement systems were highest for flexion inclination.

Our results indicate that caution should be used when attempting to infer that inclination angles measured using ViMove that are smaller than the LOA, would be the same when measured using Vicon, or vice-versa. Absolute inclination angles smaller than the LOA could be measurement error, although we cannot know from this study the sources of that error and whether they resulted from the ViMove or ViCon systems.

### How do the results compare with previous studies?

Previous studies [6, 7, 22] have considered root mean squared errors as acceptable for measuring lumbar movement if they are less than 5°, and LOA acceptable if they were less than 5° in either direction. Although these limits do not appear to have been based on empirical evidence, they do seem reasonable for the clinical assessment of lumbar region movement and are reflected in the American Medical Association’s guide to measuring spinal range of motion [23].

There are two other studies that have investigated the concurrent validity of wearable inertial systems suitable for measurement of lumbar region movements outside of the laboratory and reported root mean squared errors relative to optical motion capture reference standard systems.

Charry et al [12] investigated the validity of the wireless ViMove system for measuring *end range* lumbar region inclination motion in two participants compared with measurements from the NDI Optotrack System. Optotrack markers were placed on the surface of the sensors and they found root mean squared errors of 1° in both *end range* flexion inclination and lateral flexion inclination for single plane measurements.

Wong et.al [10] investigated the validity of measuring lumbar and thoracic region motion with a wired three-unit measurement system attached by belts at the upper trunk (T1/T2), middle trunk (T12) and pelvic (S1) level, compared with the Vicon system. *Peak value* of postural change inclination was calculated for movements in lateral flexion, flexion from neutral sitting position and stand-sit-stand for the sagittal and coronal planes in nine healthy participants. Results for differences specific to the lumbar region showed a root mean squared error of approximately 2.5° for flexion measured in the sagittal plane, and approximately 1.6° for right lateral flexion and 1.2° difference for left lateral flexion, measured in the coronal plane.

While these two studies reported root mean squared errors similar to our results, neither reported *through range* inclination measures. Clinically, the analysis of movement *through range* has the potential to identify more subtle characteristics than simple *end range*, such as the contribution and timing of components of the movement, and whether particular aspects of the movement are associated with pain.

There are other studies of the validity of wearable inertial systems suitable for measurement of lumbar region movement outside of the laboratory that have reported their findings using only correlation coefficients, *p* values and simple mean values of differences [22, 24] but these methods have been criticised as being inappropriate for comparing measurement systems [25]. We are not aware of any previous studies of lumbar region wearable inertial systems that have used the contemporary method for determining concurrent validity by estimating LOA relative to a reference standard measurement system.

### Strengths

Strengths of this study are that it was powered to be appropriately analysed with a multilevel model and that the sample consisted of a mixed population of both people with and without LBP to allow for greater variation of movement patterns. Also, other studies of wearable inertial systems and lumbar region movements have only included a healthy sample of people [10, 12, 22, 24]. We did not analyse the data separately for those participants with and without pain because clinicians assess a mixture of both types of patients, and the study was not powered to do so.

In addition, the movements that are typically tested in the clinic were measured and compared with a reference method considered accurate for surface measurements of human motion. In contrast to some earlier studies, our results were analysed and presented as average differences through the entire range of motion, instead of limiting measurements to static positions such as end range of motion, as this is how the ViMove device is likely to be used in the clinic.

Furthermore, this study is strengthened by a rigorous statistical approach based on recommended methods for measuring agreement for repeated measures, taking account of variability and autocorrelation within different levels of the data [21]. Also, we presented the results in degrees, making them directly interpretable clinically.

The reliability of sensor placement using the setup procedure that we used has been quantified elsewhere [26] and this is unlikely to have been an influential consideration in our study. That is because the current study compared two systems of measurement that independently captured movement, but both the Vicon surface markers and ViMove sensors were mounted on the same plastic frames during a single assessment session.

### Limitations

This study has several limitations. We measured differences between surface-mounted ViMove sensors and surface-mounted Vicon markers for the measurement of lumbar region movement, as this is what clinicians routinely assess in the clinic. However, this does not provide any data on the validity of ViMove sensor measurements being representative of intersegmental lumbar spine kinematic motion, as that would require a different study design and measurement methods, such as comparison with video fluoroscopy or functional MRI. When measuring lumbar region kinematics with surface-based measurement systems, some measurement error is to be expected, due to movement of skin and superficial tissues as well as the clinician’s ability to identify anatomical landmarks in a reliable and valid way [3].

Furthermore, we only included single plane movements, as these are lumbar movements typically tested in the clinic and the analysis of multi-dimensional movement involves a complexity that was beyond the scope of the current study. Similarly, as the sensors were simply calibrated to the vertical, only angles of inclination are reported.

An additional consideration is that we did not use mathematical interpolation to up-sample the ViMove data to the frequency of the Vicon data. Instead, we chose to down-sample the Vicon data to approximately 20 Hz, as we believed this to be a potentially more accurate use of the real measurements that were available. However, due to the slight variation in the sampling rate of the ViMove sensors, this method did require us to estimate the actual sampling rate for each person in each movement direction and use that rate to align the ViMove and Vicon angular data. It is possible that this method may have introduced some imprecision to estimates of differences.

### Clinical and research implications

The results of this study provide evidence of the ViMove system’s ability to measure lumbar region inclination motion with acceptable precision (concurrent validity) in a mixed sample of people with and without pain. This adds credibility to the use of this system in clinical settings to quantify lumbar region cardinal plane movement in individuals. However, it did not investigate whether complex functional movements that involve concurrent motion in several planes would affect measurement precision.

While different types of back pain may result in different patterns of movement or movement restrictions, it is not clear how this would change the average concurrent validity of the device for measuring inclination in the cardinal planes tested. Therefore, our study’s results may generalise to the broad low back pain population. However, suitably designed and powered studies would be required to test this definitively, especially for more complex 3D movement.

A recent study quantified the size of change required when using ViMove sensors to detect significant improvement above measurement error in lumbar region movement [26]. However, the potential of surface-based measurement systems to be used for measuring spinal kinematics related to treatment effects is currently unknown.

Clearly, the relatively low cost of wireless motion sensors compared to laboratory systems, and their ability to assess movement and provide individualised biofeedback in patients’ activities of daily living, are all advances that hold potential promise for innovations in the management of musculoskeletal disorders. However, exploration and validation of the clinical potential and limitations of this technology are a multi-stage and incremental process. The quantification of concurrent validity for measuring cardinal plane inclination using one type of motion sensor device is only a very early step in that process.

## Conclusion

We found clinically acceptable concurrent validity between the ViMove measurement system and the Vicon measurement system for measuring single plane lumbar inclination motion in a mixed sample of people with and without low back pain. This evidence of the ViMove system providing valid measurements of lumbar inclination means that it can be used with greater confidence to assess an individual’s single plane lumbar inclination in clinical situations and in daily activities. Further research is required to evaluate the system’s ability to measure complex functional movements and intersegmental spinal kinematics.

## Abbreviations

95%CI: 95% confidence Interval
Hz: Herz
LBP: Low back pain
LOA: Limits of agreement
